# Artificial intelligence-based cardiac transthyretin amyloidosis detection and scoring in scintigraphy imaging: multi-tracer, multi-scanner, and multi-center development and evaluation study

**DOI:** 10.1007/s00259-025-07117-1

**Published:** 2025-02-05

**Authors:** Yazdan Salimi, Isaac Shiri, Zahra Mansouri, Amirhossein Sanaat, Ghasem Hajianfar, Elsa Hervier, Ahmad Bitarafan, Federico Caobelli, Moritz Hundertmark, Ismini Mainta, Christoph Gräni, René Nkoulou, Habib Zaidi

**Affiliations:** 1 Division of Nuclear Medicine and Molecular Imaging, Geneva University Hospital, Geneva, CH-1211 Switzerland; 2 Department of Cardiology, Inselspital, Bern University Hospital, University of Bern, Bern, Switzerland; 3 Rajaie Cardiovascular Medical and Research Center, Iran University of Medical Sciences, Tehran, Iran; 4 Department of Nuclear Medicine, Inselspital, Bern University Hospital, University of Bern, Bern, Switzerland; 5 Department of Nuclear Medicine and Molecular Imaging, University of Groningen, University Medical Center Groningen, Groningen, Netherlands; 6 Department of Nuclear Medicine, University of Southern Denmark, Odense, Denmark; 7 University Research and Innovation Center, Óbuda University, Budapest, Hungary

**Keywords:** Transthyretin amyloid cardiomyopathy, Scintigraphy, Deep learning, Multi-tracer, Multi-center study

## Abstract

**Introduction:**

Providing tools for comprehensively evaluating scintigraphy images could enhance transthyretin amyloid cardiomyopathy (ATTR-CM) diagnosis. This study aims to automatically detect and score ATTR-CM in total body scintigraphy images using deep learning on multi-tracer, multi-scanner, and multi-center datasets.

**Methods:**

In the current study, we employed six datasets (from 12 cameras) for various tasks and purposes. Dataset #1 (93 patients, ^99m^Tc-MDP) was used to develop the 2D-planar segmentation and localization models. Dataset #2 (216 patients, ^99m^Tc-DPD) was used for the detection (grade 0 vs. grades 1, 2, and 3) and scoring (0 and 1 vs. grades 2 and 3) of ATTR-CM. Datasets #3 (41 patients, ^99m^Tc-HDP), #4 (53 patients, ^99m^Tc-PYP), and #5 (129 patients, ^99m^Tc-DPD) were used as external centers. ATTR-CM detection and scouring were performed by two physicians in each center. Moreover, Dataset #6 consisting of 3215 patients without labels, was employed for retrospective model performance evaluation. Different regions of interest were cropped and fed into the classification model for the detection and scoring of ATTR-CM. Ensembling was performed on the outputs of different models to improve their performance. Model performance was measured by classification accuracy, sensitivity, specificity, and AUC. Grad-CAM and saliency maps were generated to explain the models’ decision-making process.

**Results:**

In the internal test set, all models for detection and scoring achieved an AUC of more than 0.95 and an F1 score of more than 0.90. For detection in the external dataset, AUCs of 0.93, 0.95, and 1 were achieved for datasets 3, 4, and 5, respectively. For the scoring task, AUCs of 0.95, 0.83, and 0.96 were achieved for these datasets, respectively. In dataset #6, we found ten cases flagged as ATTR-CM by the network. Out of these, four cases were confirmed by a nuclear medicine specialist as possibly having ATTR-CM. GradCam and saliency maps showed that the deep-learning models focused on clinically relevant cardiac areas.

**Conclusion:**

In the current study, we developed and evaluated a fully automated pipeline to detect and score ATTR-CM using large multi-tracer, multi-scanner, and multi-center datasets, achieving high performance on total body images. This fully automated pipeline could lead to more timely and accurate diagnoses, ultimately improving patient outcomes.

**Supplementary Information:**

The online version contains supplementary material available at 10.1007/s00259-025-07117-1.

## Introduction

Misfolding of amyloid proteins leads to the accumulation of amyloid fibrils in the extracellular spaces of cardiac tissue, causing transthyretin amyloid cardiomyopathy (ATTR-CM) [1, 2]. ATTR-CM is a progressive disease that results in myocardial stiffness, which impairs cardiac function and leads to heart failure and death [2]. Patients with ATTR-CM have a poor prognosis, and when concurrent with other diseases, they further worsen the patient’s outcome [2–4]. Although ATTR-CM is an underrecognized disease, and its symptoms overlap with those of other cardiac diseases, various guidelines propose different diagnostic algorithms for ATTR-CM detection [2, 5–7]. In these diagnostic algorithms [2–7], the initial assessments for including or excluding ATTR-CM are based on medical history, clinical symptoms, echocardiography, electrocardiogram, and cardiac magnetic resonance (CMR) [2]. However, as ATTR-CM manifestations in these patients often overlap with other diseases, the final decision could not be made based only on these modalities [2]. In the final stage of diagnostic algorithms of these guidelines [2, 5–7], cardiac tissue biopsy, genetic testing, or scintigraphy should be performed to make a final decision on the presence of ATTR-CM [2].

Cardiac tissue biopsy and genetic testing are highly invasive, prone to sampling errors, and limitedly accessible [2]. Scintigraphy with amyloid avid radiotracers is becoming increasingly popular and is considered the standard for diagnosing and prognosis ATTR-CM patients [2]. The Perugini score from scintigraphy is the standard method for evaluating and scoring ATTR-CM [8, 9]. Correct Perugini grading is an important criterion for the diagnosis and prognosis of patients, which can be prone to inter- and intra-observer variability [8, 9]. Although quantitative SPECT imaging is becoming more standard, scintigraphy and SPECT analysis require extensive experience. Moreover, due to the underrecognized nature of ATTR-CM, abnormal findings in nuclear medicine departments during scintigraphy could be identified accidentally [10]. However, if these findings are not referred for amyloidosis assessment (scintigraphic examinations are mostly referred for oncology and orthopedic purposes), there is a high probability that physicians will remain unaware and miss the ATTR-CM diagnosis. Therefore, providing tools for comprehensively evaluating scintigraphy and SPECT images could enhance ATTR-CM diagnosis. Additionally, it could assist in identifying ATTR-CM cases at the processing stage referred for other indications, such as oncology and orthopedic patients within nuclear medicine departments. It could also be applied to large retrospective datasets to automatically analyze and identify patients with previously undiagnosed ATTR-CM.

Artificial intelligence-based image analysis could potentially resolve current challenges in analyzing scintigraphy. Recent research has introduced automated methods for detecting ATTR-CM from scintigraphy images [11–13], scoring Perugini grades [12], and, most recently, for the automated quantification of ATTR-CM SPECT images [14]. This study aimed to automatically detect and score ATTR-CM from total body scintigraphy images using deep learning on multi-tracer, multi-scanner, and multi-center datasets.

## Materials and methods

### Dataset

In the current study, we employed six different datasets (from 12 cameras) for various tasks and purposes. Dataset #1 consisted of ^99m^Tc-MDP bone SPECT/CT images and was used for developing the 2D-planar segmentation and localization models collected from Geneva University Hospital (HUG). Dataset #2 comprised ^99m^Tc-DPD bone scintigraphy images (total body planar (TB)) of patients suspected of ATTR, used for developing the ATTR-CM detection and scoring models, also collected from HUG. Datasets #3 (^99m^Tc-HDP, a private nuclear medicine center in Geneva, Switzerland), #4 (^99m^Tc-PYP, Rajaee cardiovascular hospital, Tehran, Iran), and #5 (^99m^Tc-DPD, Inselspital, Bern, Switzerland) included images of patients suspected of cardiac amyloidosis and were used for external evaluation from three different centers. Finally, dataset #6, a large cohort of ^99m^Tc-MDP images from 3 different centers and scanners without any labels for ATTR-CM, was used to test the capability of our model in a screening task. Two datasets, #1 and #2, were used to train and validate our models, while the remaining four datasets were used for external validation of our methodologies. Table 1 presents the summary statistics of demographic data, scanner types, and image acquisition parameters. More detailed information about each dataset is provided in the supplemental material. In the datasets for ATTR-CM detection and scoring model development and evaluation, consensus between two physicians was obtained [9]. The scoring information, based on Perugini’s grade [9], was recorded for each image in four classes, ranging from zero to three. Grade 0 indicated absent cardiac uptake and normal bone uptake; grade 1 indicated minimal uptake in the myocardium, less than that in bone tissues; grade 2 indicated uptake in the myocardium equal to that in bone tissues; and grade 3 indicated uptake in the myocardium higher than that in bone tissues [9]. In summary, datasets #1 and #6 were collected from patients referred for bone scintigraphy to evaluate bone pathologies, including benign and malignant lesions. While datasets #2, #3, #4, and #5 include patients suspicious of ATTR-CM referred for evaluation of cardiac amyloidosis.

**Table 1:**
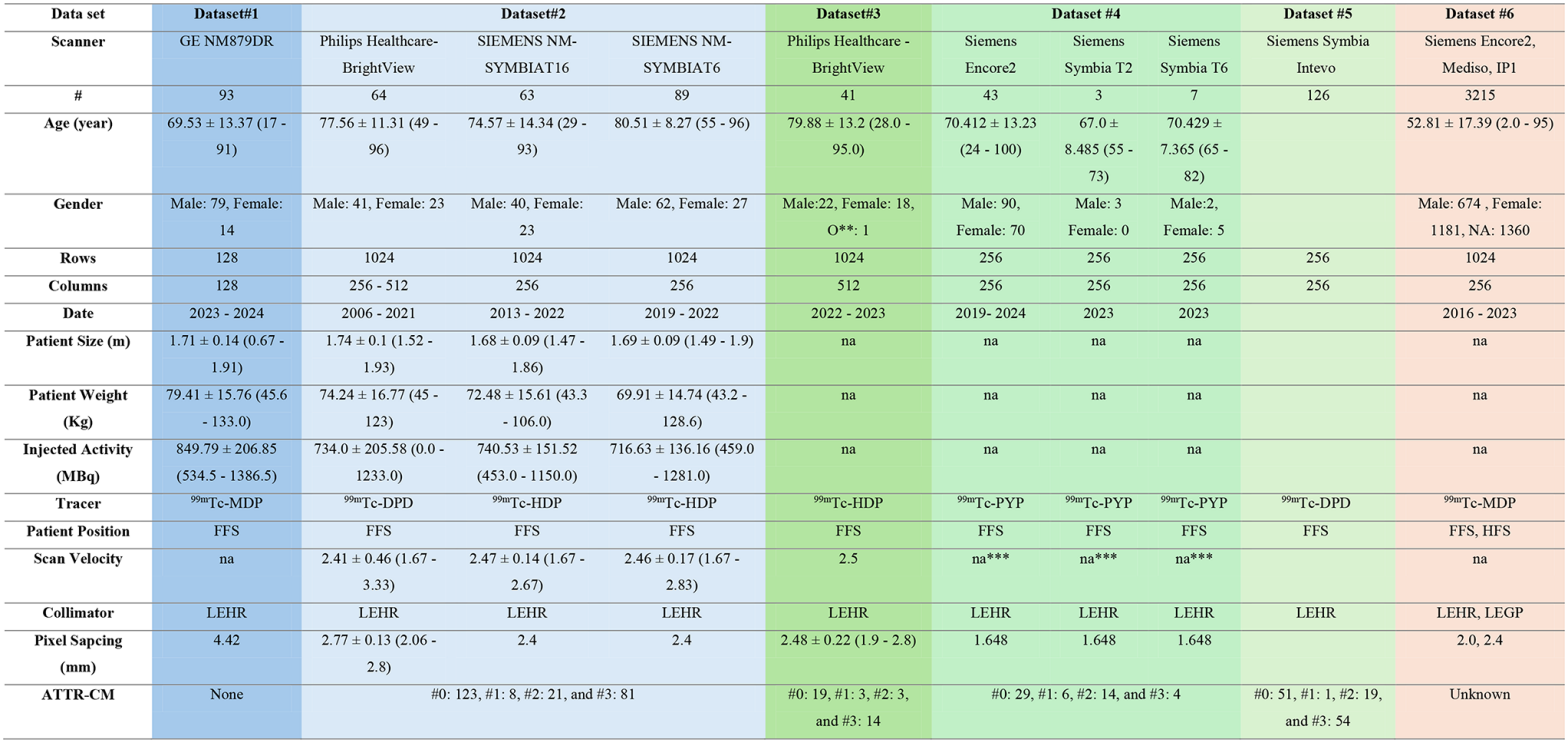
Dataset information and demographics. * refers to ^99m^Tc-MDP scans for bone pathologies. **: unknown gender, ***: these images were spot static planar scans with a fixed bed position. Datasets 1 and 2 were used for model development (blue), datasets 3–5 were used for external salvation (green), and dataset 6 was used for screen purposes (orange), which is data without any ground truth. LEHR: low-energy high-resolution collimator. LEGP: low-energy general purpose collimator. FFS: feet first Supine. HFS: Head First Supine

To create a fully automated pipeline for detecting and scoring ATTR-CM in scintigraphy images, we proposed two main steps: automatically detecting and localizing the region of interest, followed by classification for detection and scoring of ATTR-CM. In the following, we explain each step in detail.

### Region of interest detection on TB planar images

**Fig. 1:**
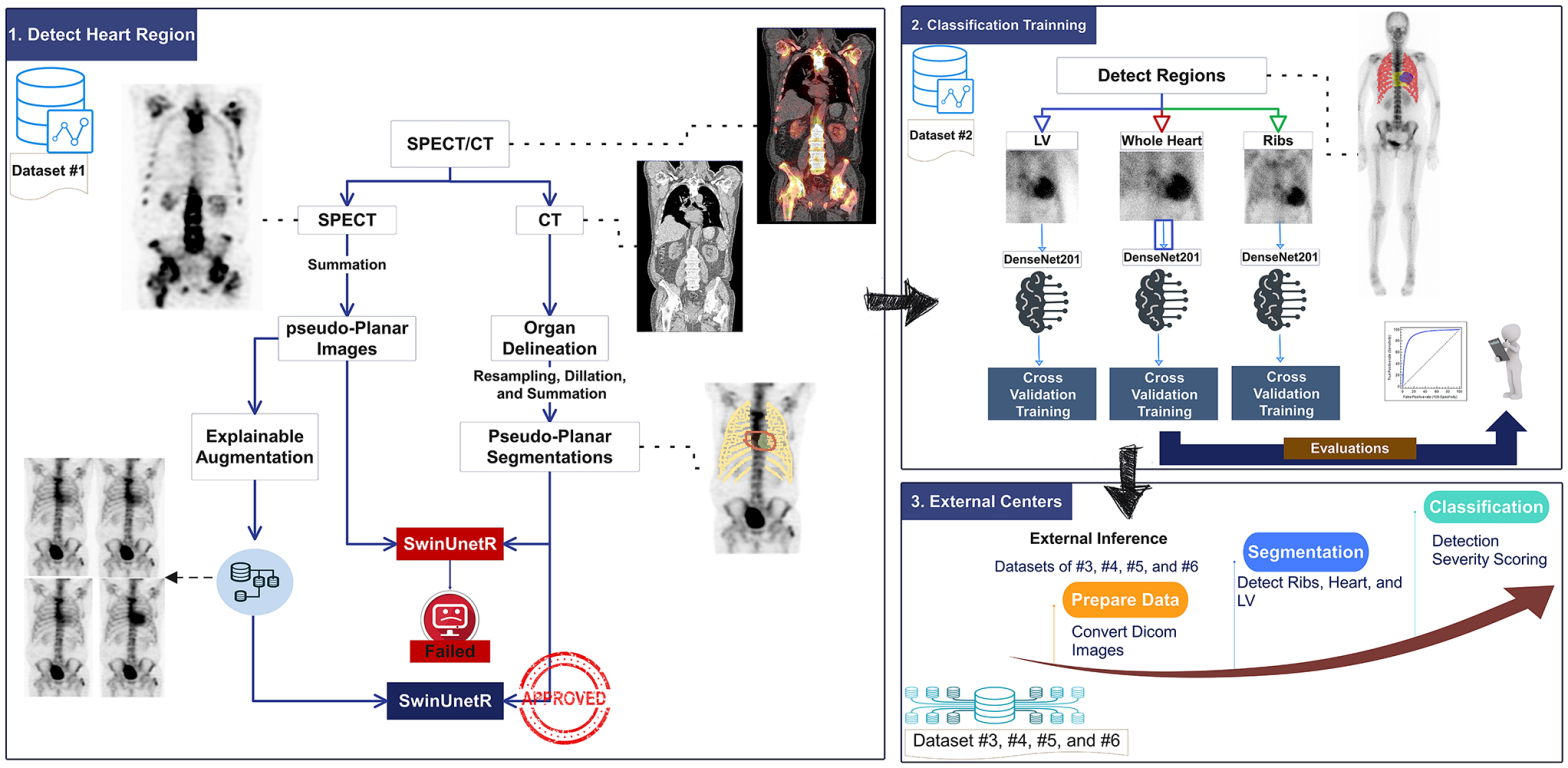
Workflow depicting the whole pipeline followed in this study. Each step was performed using a different database. Step (1) Detect Heart Region was trained using dataset #1, the first attempt using original pseudo-planar images from dataset #1 was unsuccessful as shown with the sign “failed” in the diagram; however, the performance was improved after explainable data augmentation. step (2) Classification training using dataset #2, and the developed models were tested in step (3) External centers on datasets #3 to #6

TB planar images capture the total body, while cardiac information is limited to the chest and cardiac areas. To develop a robust and fully automated ATTR-CM detection and scoring model, we cropped TB images before feeding them into our deep-learning classification model. To achieve this, we created a model using deep learning 2D segmentation to detect the whole heart, left ventricle (LV), and ribcage area. Figure 1 summarizes the automated heart detection process on TB planar images pipeline.

#### TB image/segmentation ground truth generation

Due to the absence of segmentation in TB images, using a multiple-step strategy, we tried a hybrid method to generate the ground truth image/segmentation pair of planar images. SPECT/CT images of ^99^Tc-DPD images were limited to the cardiac region, while we needed image/segment pairs covering the whole thorax area. Hence, we moved to ^99^Tc-MDP images with almost the same distribution and uptake in bones, kidneys, and urinary bladder. First, we enrolled 93 thoraco-abdominal bone SPECT CT images injected with ^99^Tc-MDP from dataset #1). Of 93 cases, 8 cases were excluded due to small coverage in the cranio-caudal direction. Second, a few selected organs of the whole cardiac LV and ribcage were segmented through CT images using pre-trained models developed in our group [15]. These segmentations were co-registered with SPECT images, dilated by 2 mm and resampled to be in the same space as SPECT images. SPECT images were summed over anterior to posterior direction to generate a pseudo-planar image covering the thorax, abdomen, and pelvis regions as described before [16].

There is no magnification in planar gamma camera imaging using parallel whole collimators, and SPECT/CT segmentation is at the same size as planar images. The same transform was implemented on the 3D ribcage, whole heart, and LV segmentation to generate 2D pairs of pseudo-planar images and segmentation masks. These images were used to train three different deep learning-based segmentation models to segment ribs, whole hearts, and LV. Finally, we inferenced the segmentation models on 2D TB images on other datasets.

#### ATTR-CM pseudo-planar simulation

It should be emphasized that these training images were from images suspected of bone pathologies and with no ATTR history, i.e., there was no tracer uptake in the myocardium, same as class #0 TB images from datasets #2 and external datasets #3, #4, and #5. At the first training attempts, the segmentation models were good enough on validation images from dataset #1 and class #0 cases from dataset #2; however, the model performance on TB planar images with absorption in cardiac tissue was not acceptable as the model only saw the normal cases during training.

To overcome this limitation, we generated a pseudo-planar and segmentation population with the simulated cardiac amyloidosis pattern by adding various uptake intensities inside the LV segmentation. The LV segmentation was already co-registered with pseudo-planar images. We masked a random image between a certain range of values ranging from 5 to 150% absorption of the 99% percentile of the image in a 5% step. After this step, each image was augmented to 29 images corresponding to a range from normal (class #0) to severe cardiac uptake with an uptake 1.5 times more than the 99 percentile of the image (class #3) by adding the random LV distribution and the pseudo-planar image. The initial number of 85 cases was converted to 2465 cases through offline data augmentation. Each image was augmented 28 times by adding different simulated uptake to the cardiac area and saved locally. The augmented dataset was used to retrain the segmentation models, seeking a robust model for TB planar segmentation and cardiac area detection. Supplementary Fig. 4 presents examples of augmented images for a pseudo-planar image corresponding to different levels of uptake in the heart. It should be noted that these images were used only for the segmentation step to develop a reliable image segmentation model. SPECT and pseudo-planar images had 128 × 128 matrix size, and they were resampled to 2.8 × 2.8 mm^2^ isotropic resolution before being used as input to segmentation models.

#### Data preparation

The MONAI platform was used to train deep learning segmentation models using SwinUnetRV2 network architecture, 2.8 × 2.8 mm^2^ pixel dimensions, 2D patch size of 256 × 704 pixels, and sliding window inference with 50% overlap between the patches. The images were normalized between zero and one by clipping input values between zero and 99 percentiles of the input image; then training was continued for 100 epochs with a batch size of 2, an initial learning rate of 1e-4 reduced piecewise and minimizing Dice cross-entropy loss. Finally, the best validation model was saved. Three separate models were trained for segmentation of LV, heart, and ribcage, and the best models were saved for inference on TB planar images.

None of the 2D scintigraphy and 3D SPECT images are quantitative, and the voxel value depends on acquisition time, delay after tracer injection, and the amount of tracer administrated to the patient. In this study, we normalized every SPECT image to its own 99 percentile of the image distribution to harmonize the variations in the voxel value range, which is not negligible. This approach is applicable to any external image from any scanner as each image would be normalized to its own 99 percentile, and both classification and segmentation outputs are binary values that are not affected by the normalization method. Moreover, we used 99 percentiles instead of maximum value to eliminate the effect of high voxel values related to image noise in SPECT images.

TB anterior and posterior images were summed together after flipping the posterior image from the right to the left axis to generate an image with better statistical noise, closer to the pseudo-planar images. This image, called the summation image, was saved and used in further steps. The ribcage, LV, and whole heart were segmented on TB planar images using three separate models. The LV, whole heart, and ribs segmentation were separately cropped by the bounding box covering the 70-, 50-, and 1-mm margins, respectively.

### Deep learning classification

#### Data preparation

Due to the highly imbalanced nature of the four classes, we defined two classification tasks, including the detection task, where images in class #0 were separated from images in class #1, #2, or #3 (grade 0 vs. grades 1, 2, and 3), and the severity scoring task, to discriminate between images in classes #0 and #1 and classes #2 and #3 (0 and 1 vs. grades 2 and 3). Using the previous automated region of interest detection on the TB images pipeline, three sets of cropped images were generated and used to train three different classification models; then, the results were compared. All patient images from data set #2 were randomly split into three folds, and at each training fold, 2/3 of the data was used for training and validation, and 1/3 of the data was used as an unseen test dataset. It should be mentioned that we made sure that for patients with more than one acquisition, all images were in the same teat or train set (patient-wise data splitting).

#### Model development

All cropped TB-planar images were fed to a DenseNet 201 network architecture after being resampled to 2.8 × 2.8 mm^2^ pixel spacing and normalized between zero and one using 99 percentiles of the image as the maximum. Offline augmentation, including random rotation, adding noise, and flipping, was implemented on the training and validation images separately for each data class to gain a balanced augmented number of cases in each class.

Model training was continued for 200 epochs with an initial learning rate of 1e-4 reduced piecewise with 95% decay and cross-entropy loss function while training different parameters of F1-Score, sensitivity, AUC, specificity, and accuracy were calculated, and the best average metric model saved. The model performance was characterized using common classification metrics, including F1-Score, sensitivity, AUC, specificity, BAC (balanced accuracy defined as the average of sensitivity and specificity), accuracy, and receiver operative curve (ROC). This three-fold classification training was repeated for every three cropping strategies of crop to ribs, whole cardiac, and left ventricle. The overall performance was compared between nine possible combinations of models, which were the product of three input images (anterior, posterior, and summation) and three cropping strategies (LV, whole heart, and ribcage).

#### Model ensemble and interpretability

Automated cropping strategies were used to detect the cardiac region. Then, the results of the three-fold models trained on the corresponding cropping strategy were inferred from the cropped images. The results from the three folds were combined by averaging the probabilities and selecting the class with the higher probability through ensemble averaging. Each model’s output after the Softmax layer is a probability value between 0 and 1, which is then converted to a binary class after activation. In our approach, the probabilities from all models were averaged, and the final decision was based on the average probabilities. For example, consider the outputs of three different models with two classes (class #0 vs. class #1) and the following probabilities: 0.45 vs. 0.55 (predicted class #1), 0.95 vs. 0.05 (predicted class #0), and 0.48 vs. 0.52 (predicted class #1). Voting among these three models would result in predicted class #1, as two of the models predicted class #1. However, probability averaging would predict class #0, since the second model has a significantly higher probability and indicates that the predicted class carries a higher certainty. The average probabilities are (0.45 + 0.95 + 0.48) ÷ 3 = 0.627 for class #0 and (0.55 + 0.05 + 0.52) ÷ 3 = 0.373 for class #1, so the predicted class using averaging the probabilities would be class #0. This approach may outperform voting methodologies by considering not only the predicted class but also the uncertainty in each model’s output.

**Fig. 2:**
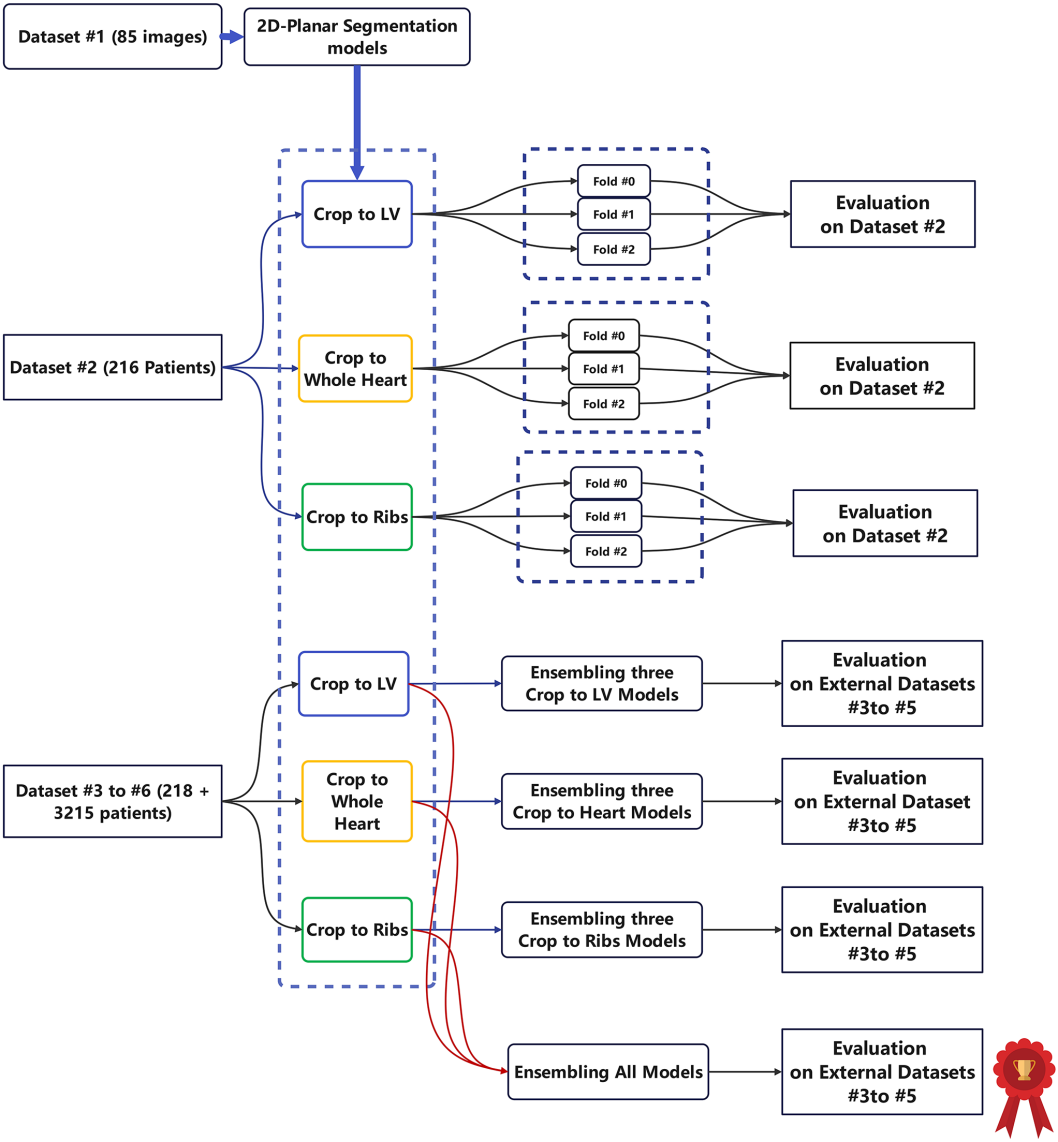
Data flow in this study. Dataset #6 was used for inference, but this was not included in the evaluation phase. The external data evaluations were performed using all models ensembled as shown by a red sign

The same classification metrics were measured. Two steps of averaging probabilities were implemented; first, for each crop strategy, three models trained for each fold were tested on a single image, and the average probability was used to decide; second, the averaged three-fold probabilities from each crop strategy were averaged and used for final decision using all folds and crop strategies. Figure 2 summarizes the steps followed in this study for training the classification models and external validation. In addition, to make the decision-making process more explainable and check the validity of the decision, the importance of GradCam map and Occlusion Sensitivity maps [17] were generated for all images.

### External centers

Datasets #3, #4, and #5, containing 218 cases (41, 53, and 126) from 5 scanners, were used as external unseen datasets. Dataset #6, without the ground truth for ATTR-CM, was used to evaluate the performance of our pipeline in a real-world scenario. This large dataset, including 3215 total body planar images for patients injected with ^99^Tc-MPD, were acquired from multiple centers and scanners and acquisition protocols [18]. There was no cardiac amyloidosis indication for these patients, and we had no information about the presence or absence of cardiac uptake, i.e., there was no Perugini score available, as this was the case for the other external datasets mentioned above. In total, all 27 models including three folds, three crop strategies, and three image input were inferenced on each external image and the final decision was made by considering all models through ensembling probabilities.

In summary, we developed two separate deep learning models: first a segmentation model to detect the cardiac region and cropping the images and second, a classification model to classify the cropped images. We defined two classification tasks, the first detection task separates the images in class #0 from the rest (#1, #2, or #3), while the second severity scoring task separates #0 and #1 classes from severe uptake in classes #2 and #3. We used innovative explainable data augmentation methods to have a robust segmentation model on planar images with different patterns of uptake in the heart. In addition, we trained a separate classification model for images cropped to the whole heart, left ventricle, and ribcage. The final decision about each external case was made by considering the decision and the uncertainty in the decision made by each single model. We tested our model on four external datasets from other centers; these images were new to the segmentation and classification models.

## Results

### Region of interest detection on TB planar images

**Fig. 3:**
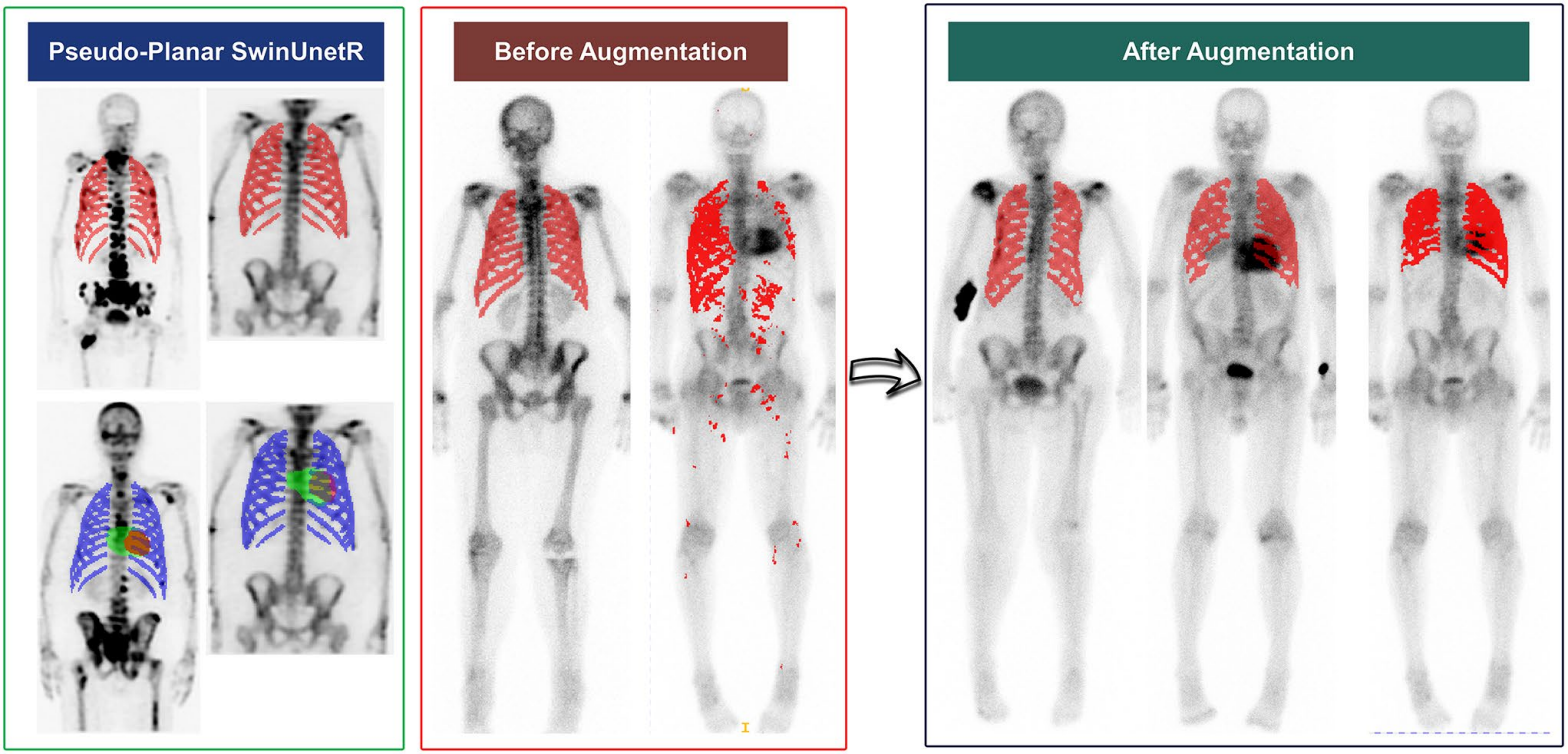
Examples of segmentation output on the pseudo-planar images as well as total body planar images segmented with SwinUnetR before and after explainable data augmentation

**Fig. 4:**
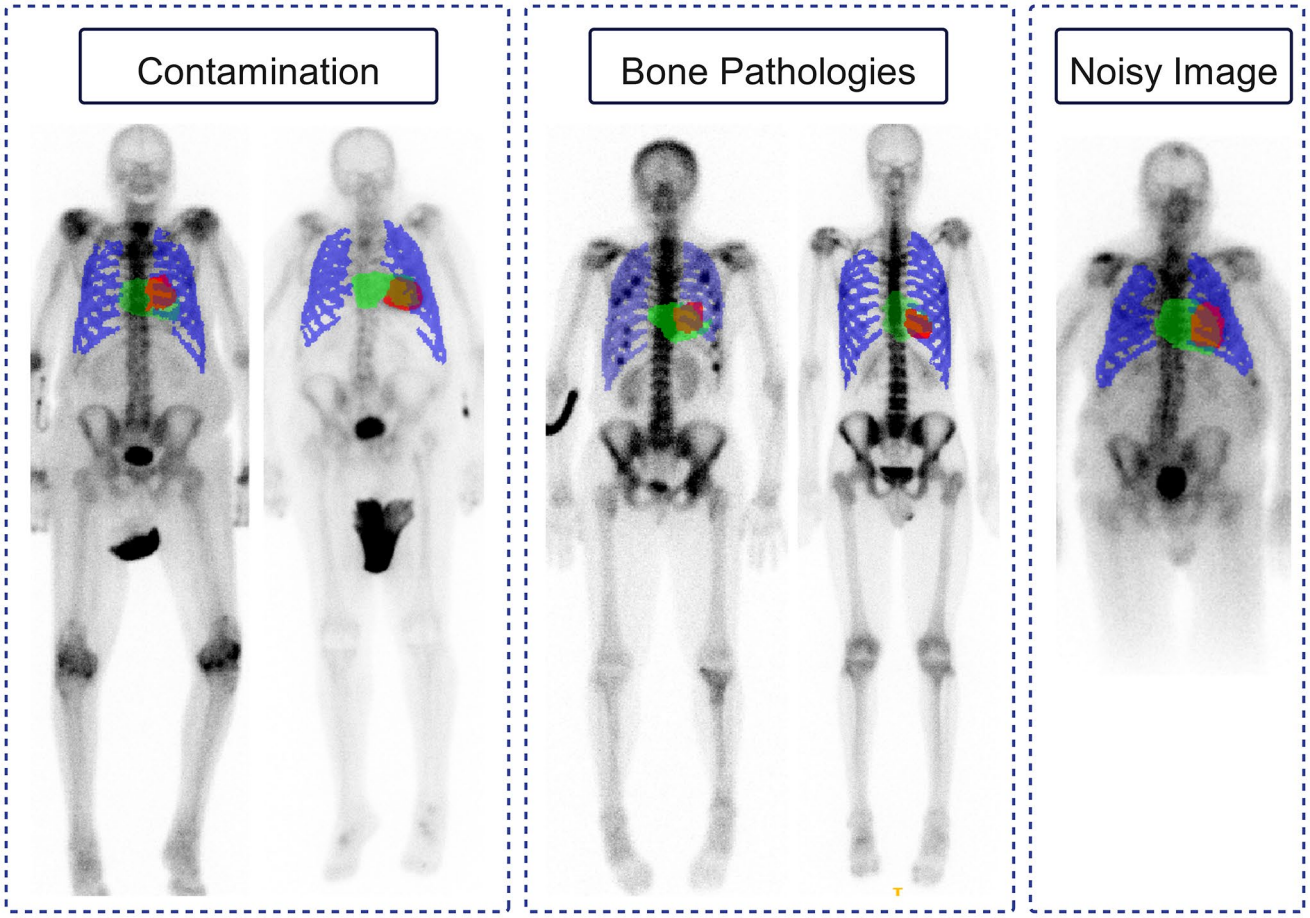
Segmentation of a few challenging cases with urine bags or contaminations

The segmentation Dice score on pseudo-planar images was more than 99.9% for all three segments: the ribcage, LV, and the whole heart. Figure 3 displays examples of pseudo-planar image segmentation, as well as segmentation before and after data augmentation for the ribcage in several cases. After augmentation, all cropping areas included the cardiac region except for two cases. Figure 4 shows the acceptable performance of our cropping method on a few challenging cases with uptake in a urine bag, noisy image, and a patient with hot uptake points at the ribcage.

Supplementary Fig. 1 presents examples of outliers where the segmentation accuracy is low. However, the cropping area is unaffected because the bounding box covering the segmentation includes the cardiac area, except for one case. In this exception, there is extensive extravasation and the presence of a radiotracer outside the body. The main reason for the segmentation outlier was the low contrast-to-noise ratio on the TB image.

### Deep learning-based classification

#### Internal test set

**Fig. 5:**
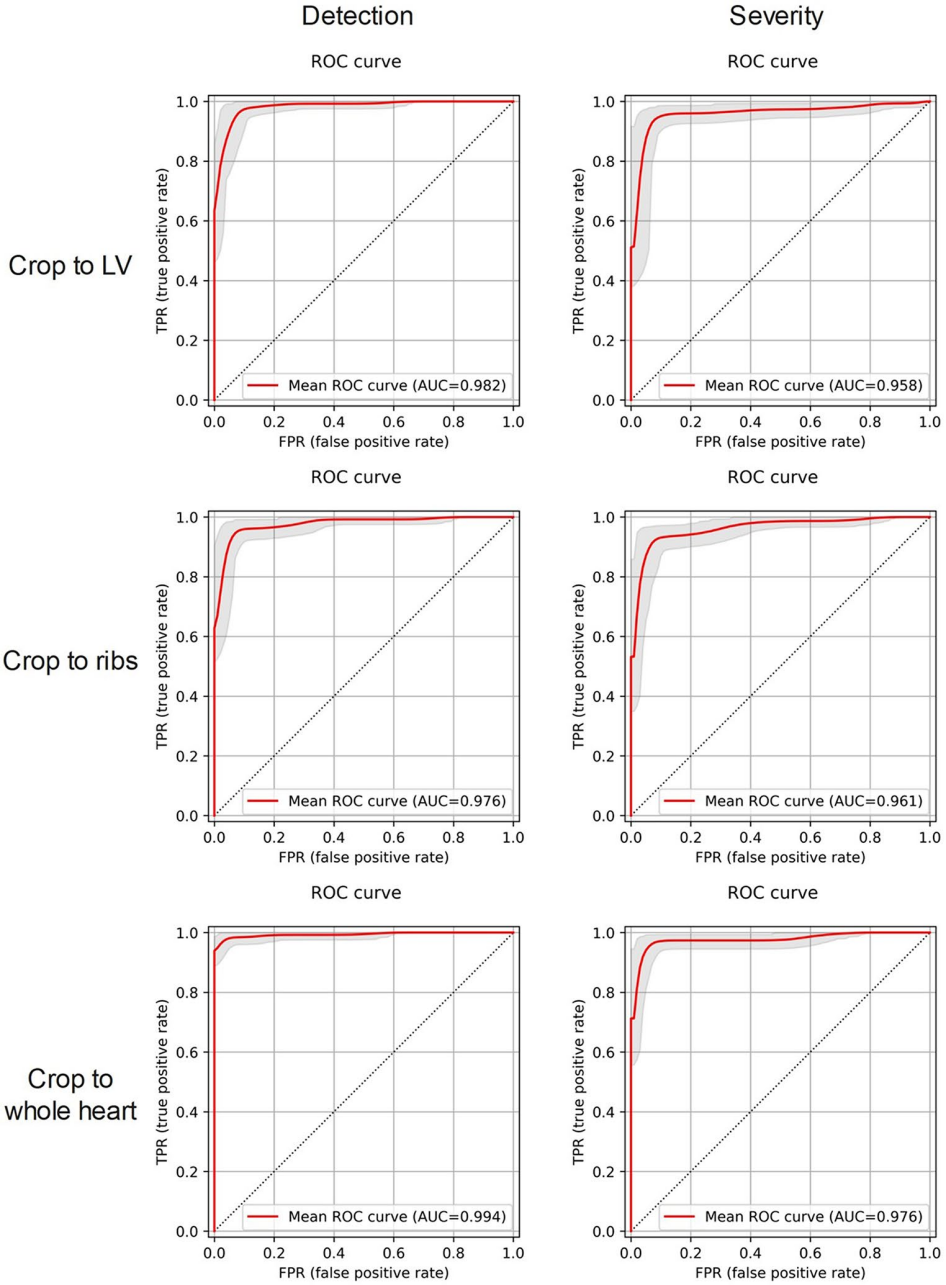
Three-fold internal cross validation ROC curves for three cropping strategy and two tasks

The overall accuracy over three folds for three cropping strategies and three input images was summarized in Table 2. The detection task’s highest values were slightly higher than the severity-scoring tasks. The best detection task performance was achieved using an anterior image cropped to the whole heart, with sensitivity, specificity, precision, accuracy, BAC, and AUC of 0.969, 0.981, 0.984, 0.974, 0.975, and 0.995, respectively. Figure 5 shows the ROC curves for the best model for detection and severity scoring of ATTR-CM, which is averaged over three folds. The normalized confusion matrix is presented in Supplementary Fig. 2.

**Table 2:** Summary of classification three-folds results. BAC: Balanced Accuracy. AUC: Area under the curve

Task	Crop	Input	F1	Sensitivity	Specificity	Precision	Accuracy	BAC	AUC
Detection	LV	Anterior	0.957	0.961	0.942	0.953	0.952	0.951	0.982
		Posterior	0.929	0.922	0.922	0.937	0.922	0.922	0.958
		Summation	0.941	0.938	0.932	0.945	0.935	0.935	0.969
	Ribs	Anterior	0.935	0.961	0.883	0.911	0.926	0.922	0.972
		Posterior	0.943	0.961	0.903	0.925	0.935	0.932	0.981
		Summation	0.911	0.914	0.883	0.907	0.900	0.899	0.954
	Whole heart	Anterior	0.976	0.969	0.981	0.984	0.974	0.975	0.995
		Posterior	0.929	0.914	0.932	0.944	0.922	0.923	0.974
		Summation	0.957	0.945	0.961	0.968	0.952	0.953	0.978
Severity	LV	Anterior	0.943	0.933	0.916	0.952	0.927	0.924	0.952
		Posterior	0.946	0.927	0.940	0.965	0.931	0.933	0.956
		Summation	0.953	0.947	0.928	0.959	0.940	0.937	0.962
	Ribs	Anterior	0.943	0.940	0.904	0.946	0.927	0.922	0.952
		Posterior	0.932	0.920	0.904	0.945	0.914	0.912	0.943
		Summation	0.949	0.927	0.952	0.972	0.936	0.939	0.965
	Whole heart	Anterior	0.956	0.940	0.952	0.972	0.944	0.946	0.980
		Posterior	0.938	0.913	0.940	0.965	0.923	0.927	0.950
		Summation	0.966	0.960	0.952	0.973	0.957	0.956	0.977

#### External test set

**Fig. 6:**
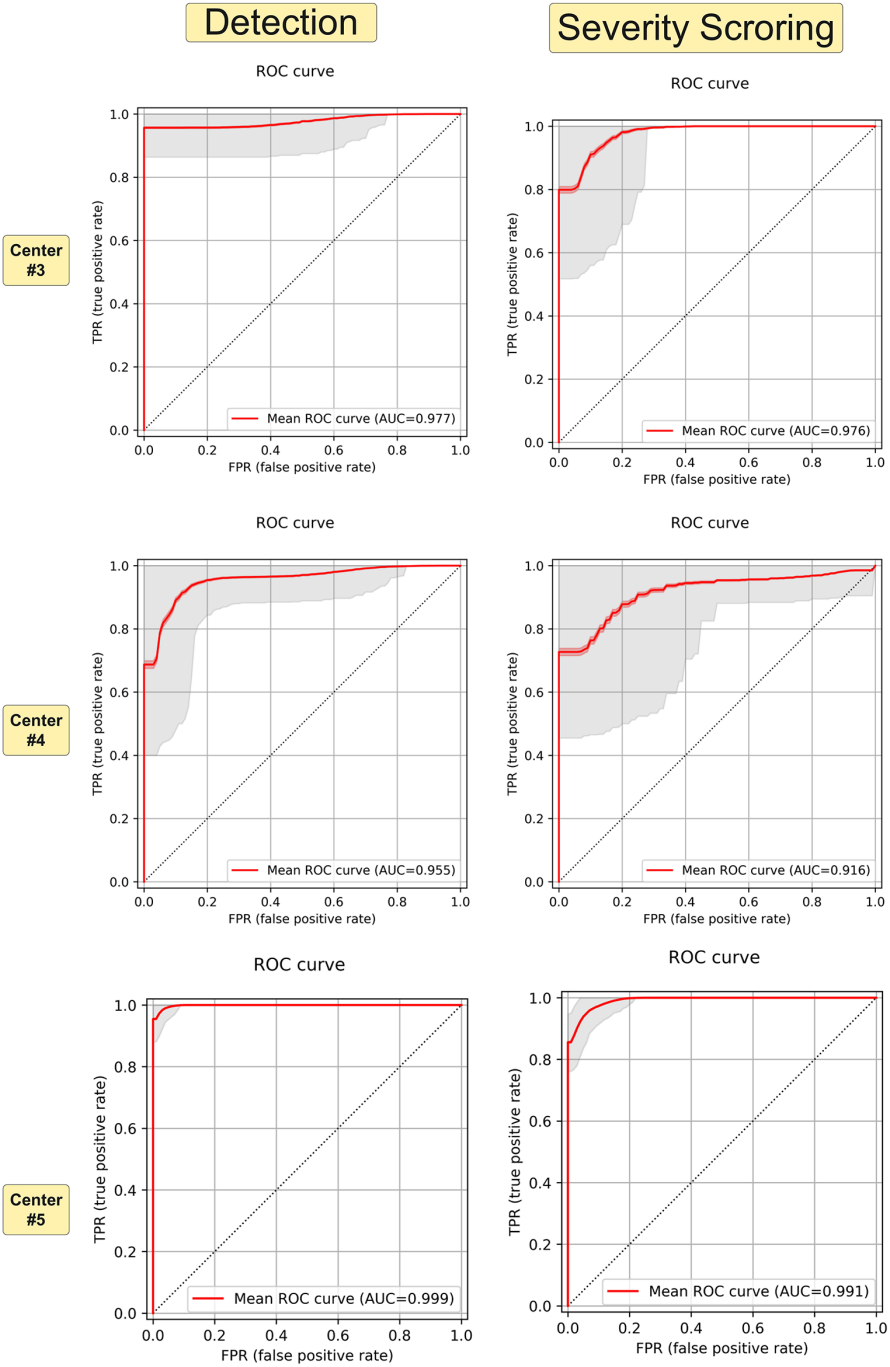
ROC curve for external dataset #3, #4, and #5

Table 3 summarizes the performance metrics on the three external datasets #3, #4, and #5. The best accuracy of 1 (100%) was achieved for dataset #5, and the lowest performance was observed on dataset #4, especially in the severity detection task. This dataset was acquired using different acquisition protocols as the training data. Figure 6 depicts ROC curves for these three external validation datasets. Supplementary Fig. 3 presents the normalized confusion matrix for the same approaches.

**Table 3:** Accuracy results on the external datasets of #3, #4, and #5 for the ensembled models. BAC: Balanced Accuracy. AUC: Area under the curve

Center	Task	Input image	Crop strategy	#	F1	Sensitivity	Specificity	Precision	Accuracy	BAC	AUC
Data set #3	Detection	All Three	All Three	39	0.958	0.958	0.933	0.958	0.949	0.946	0.978
	Severity	All Three	All Three	39	0.962	0.962	0.923	0.962	0.949	0.942	0.964
Data set #4	Detection	All Three	All Three	53	0.947	0.931	0.952	0.964	0.940	0.942	0.956
	Severity	All Three	All Three	53	0.964	0.952	0.875	0.976	0.940	0.914	0.889
Data set #5	Detection	All Three	All Three	126	0.985	0.985	0.989	0.985	0.987	0.987	0.999
	Severity	All Three	All Three	126	0.948	0.970	0.943	0.928	0.954	0.956	0.992

#### External test set without the ground truth

**Fig. 7:**
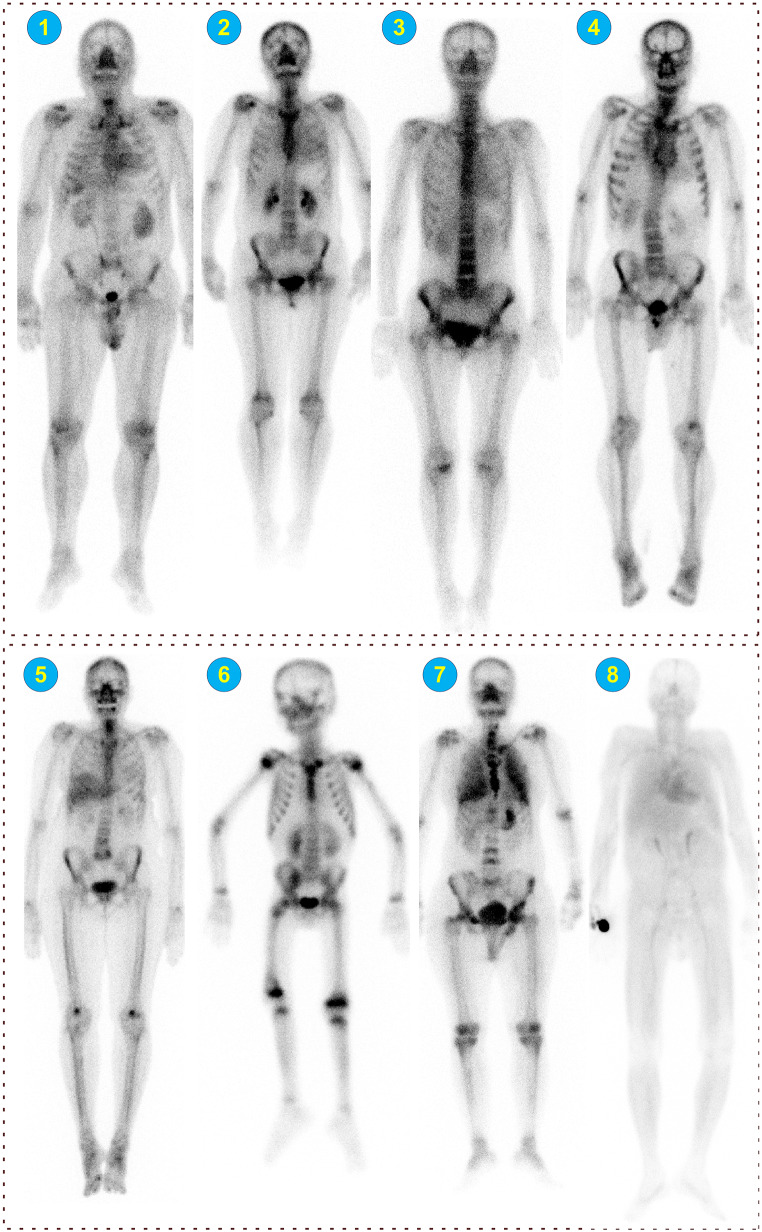
External cases from dataset #6, the top row (1 to 4) shows images that interpreted as needing more cardiac evaluation, whereas the bottom row shows cases detected by our model as false positives. Case #5: an image with liver uptake, #6: a pediatric case, #7: lung uptake, and #8: a blood pool image

From 3215 images, ten images were detected as positive for cardiac amyloidosis uptake. Images were checked by a nuclear medicine physician, and four of them were confirmed to be susceptible to unusual cardiac uptake. Figure 7 presents examples of detected cases from the large ^99m^Tc-MDP dataset (dataset #6). The top row shows cases detected and verified to be susceptible to cardiac amyloidosis, and the bottom row shows the false positive cases.

### Model interpretability

**Fig. 8:**
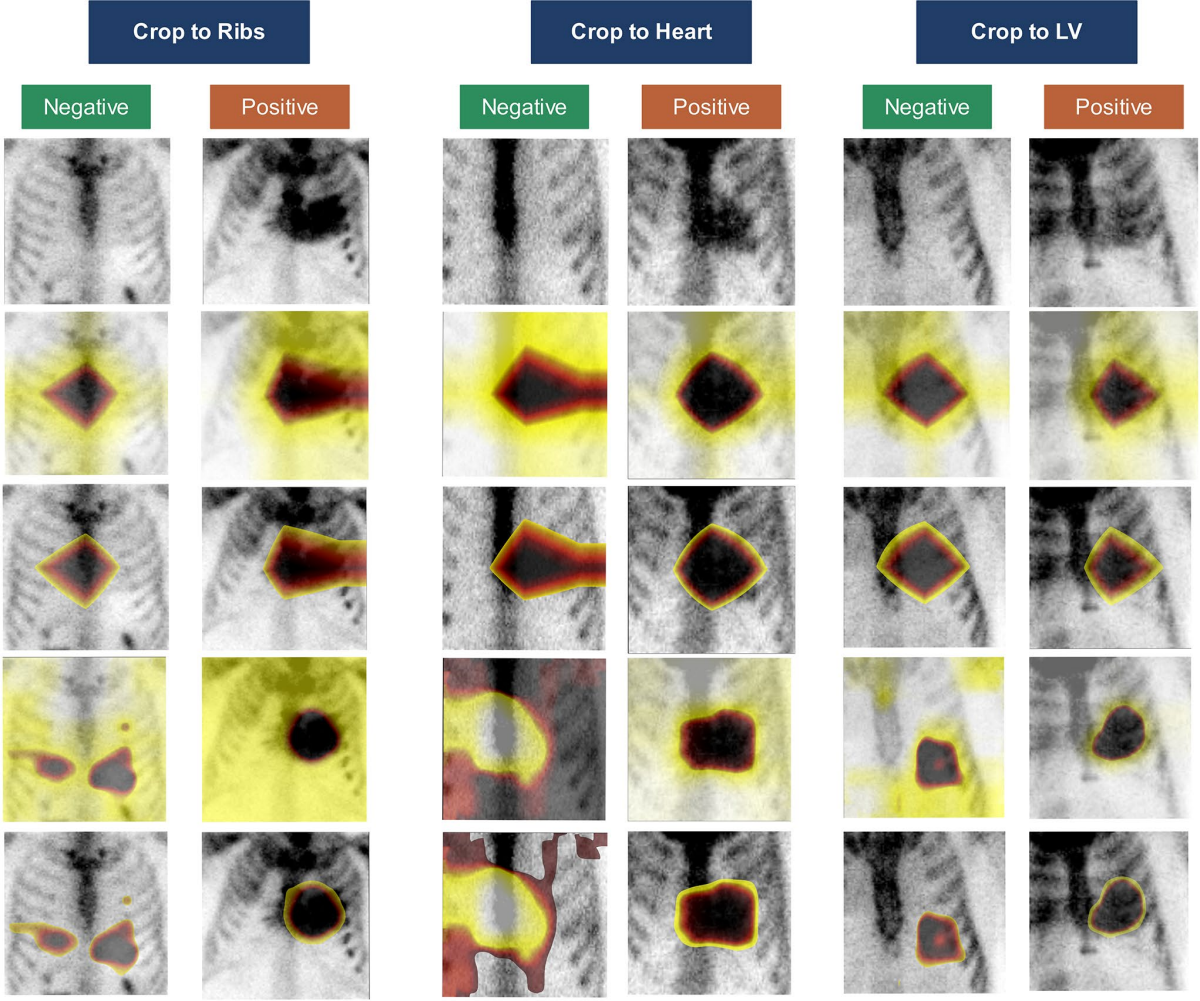
GradCam and saliency map inference showing the area on which the classification deep learning models were focused. From top to bottom row: TB cropped image, GradCam map as an overlay, GradCam map with a threshold as an overlay, Saliency map as an overlay, Saliency map with a threshold as an overlay

Figure 8 shows the GradCam and saliency map generated during inference for models trained on images with different crop strategies, both for positive and negative cases. The saliency map shows the focused area in more detail than the GradCam. For most cases, the focused area was around cardiac, chest, and clinically relevant regions.

## Discussion

ATTR-CM progresses as amyloid proteins misfold and accumulate in the cardiac tissue, leading to myocardial stiffness, impaired heart function, and, ultimately, heart failure and death [2–7]. Despite its critical impact, ATTR-CM is often underrecognized because its symptoms overlap with those of other heart diseases, making diagnosis challenging [2–7]. Diagnostic protocols suggest initial evaluations with non-invasive techniques, such as echocardiography, ECG, and CMR, but definitive diagnosis often relies on more invasive methods, such as tissue biopsies or genetic testing, which have their own limitations and associated risks [2–7]. In recent years, the use of amyloid-avid radiotracers and the Perugini scoring system have revolutionized the diagnosis and prognosis of ATTR-CM [2–7]. Nonetheless, the assessment of these imaging techniques requires significant expertise [2–7]. AI-based image analysis provides automated tools for more accurate detection and quantification of ATTR-CM from imaging studies to address the existing issues in scintigraphy and SPECT image analysis. This integration of AI promises to enhance diagnostics by efficiently processing large datasets and reducing human errors.

In this study, we developed a fully automated pipeline to detect and score ATTR-CM from scintigraphy images. TB planar images capture the total body, while cardiac information is limited to the chest and cardiac areas. Deep learning classification algorithms may focus on information outside the clinically relevant area and still perform well in separating different groups [19]. To have a robust and trustworthy classification model, we cropped the TB images before feeding them into our deep-learning classification model. This ensured the model was focusing on the clinically relevant area. Additionally, we aimed to generate a fully automated pipeline for planar image classification. To achieve this, we developed a separate model to detect the chest area using deep learning 2D segmentation. To develop the segmentation model, we used CT image segmentation transferred to pseudo-planar images as ground truth, transferred it to TB images, and generated and simulated different uptake levels of cardiac regions representing different grades of ATTR-CM to have a realistic explainable data augmentation. Augmentation could resolve the differences between training images from dataset #1 and datasets #2 to #5. SPECT and CT images are co-registered and well-aligned, with the location information stored in the DICOM header. Unfortunately, this is not the case for SPECT and total-body planar scintigraphy images. As a result, the segmentation masks generated from CT images are not co-registered with total-body planar bone images, necessitating the use of pseudo-planar images.

After cropping the images to different regions, we built two different models for detection and severity scoring. These models were evaluated both internally and using external center datasets. Our pipeline showed the best performance on external dataset #5, even though it used a different tracer, both the detection and severity scoring models performed well, likely due to the excellent image quality of this external dataset. The lowest performance was observed with center #4, likely due to low-quality images produced by an older scanner, different imaging technique (spot planar vs. continuous bed motion TB planar in training), and the different tracer. The interpretability of the models showed that both models are focusing on the relevant regions to correctly detect and score ATTR-CM in TB images in all cropping strategies. Moreover, we applied the fully automated pipeline to a large dataset from different centers without any ground truth for ATTR-CM. Out of this large dataset, we found ten cases flagged as ATTR-CM by the network. Out of these, four cases were confirmed by a nuclear medicine specialist as possibly having ATTR-CM and suggested more evaluation to roll out ATTR-CM. This demonstrates the performance and capability of this model to be applied retrospectively to large datasets and identify new cases as a screening tool. Our model identified ten images from 3215 total body bone scintigraphy images as ATTR-CM positive. Figure 7 (bottom row) shows 4 of those cases and an explanation on why they were falsely detected by our model. It should be noted the NM physician did not go through all 3215 images, which is very time-consuming. Hence, there might be cases suspicious for cardiac uptake and not detected by our model that we are not aware of. We introduced a fully automated classification method including two steps of region detection through segmentation and classification. In order to explain the contributions of each step in the final performance, we visualized the regions detected by our segmentation models on dataset #3 and the results are presented in supplementary Fig. 5. Of 41 images, LV was inaccurately segmented on three images and the whole heart on one image. It should be noted that in all four images, the other two cropping approaches were approved. The final results and decision on an external image depend on the decision made by all three cropping strategies to reduce the effect of false region detection in cases similar to those indicated with a red box in supplementary Fig. 5.

We ensembled all 27 models on each external image. These results and approach tend to be more reliable and reproducible on external unseen cases as it considers the bias, strength, and weakness of all training subsets. Although the best results in cross-validation training on dataset #2 was achieved using anterior images, but the other two images were acceptable. The higher accuracy using anterior images was expected as there is less information in the posterior projections due to ribs and vertebrae attenuation effect. We generated GradCams and saliency maps and our models showed excellent focus on clinically relevant areas, still the combination of models through ensmbeling probabilities was a safer ground to be tested on the external datasets and to be shared with other groups for testing.

Various studies were carried out to detect ATTR-CM in different imaging modalities, including scintigraphy [2–7]. Halme et al. [12] developed a DL model to automatically identify ATTR-CM from ^99m^Tc-HMDP scintigraphy of 1,334 patients (47 ATTR-CM). They attempted to classify ATTR-CM according to Perugini grades, specifically comparing grades less than 2 versus 2 and higher and grades less than 3 versus grade 3. The models were validated through 5-fold cross-validation and achieved an AUC of 0.87 for multiclass classification, 0.94 for the binary comparison of grades less than 2 versus grades 2 and higher, and 0.89 for grades less than 3 versus grade 3. Delbarre et al. [13] used ^99m^Tc-DPD/^99m^Tc-HMDP scintigraphy of 4,681 patients (383 ATTR-CM) for automated detection of patients with Perugini grades higher than two. The DL model was trained using 3,048 patients and evaluated using external validation testing on 1,633 patients, who reported an AUC of 0.99 using the internal and external test sets.

In a multinational study by Spielvogel et al. [11], a DL system was developed to standardize and enhance the reliability of screening for ATTR-CM suggestive uptake using 99mTc scintigraphy data from multiple tracers and scanners. The cohort included 16,241 patients, with 15,808 showing non-cardiac amyloidosis-suggestive uptake (Perugini grade 0 or 1) and 433 indicating cardiac amyloidosis-suggestive uptake (Perugini grade 2 or 3). They reported diagnostic accuracy with an AUC of 0.92-1.0 across several cohorts and centers. They compared the performance of the DL system with physicians; the DL system outperformed human physicians in a multi-center, multi-reader study, demonstrating significantly less inter-rater variability and higher predictive performance. Furthermore, the AI predictions were also prognostic of overall mortality. We achieved the same performance as Spielvogel et al. [11] study in multi-tracer, multi scanner, and multicenter settings for ATTR-CM detection. Our study introduces a novel automated preprocessing step, contributing to a fully automated pipeline for detecting ATTR-CM. We introduced an explainable data augmentation technique to improve the detection accuracy. We introduced three cropping approaches, where each model was trained in cross-validation training and 27 different models were inferenced on an external case to have a more robust model through ensemble averaging. All models showed a good focus on clinically relevant areas proved by GradCam and saliency maps. Furthermore, we made the model publicly available, enabling broader research utilization and supporting future advancements in clinical applications.

Miller et al. [14] utilized DL algorithms to automate the volumetric quantification of single center ^99m^Tc-pyrophosphate SPECT/CT images in patients suspected of having ATTR-CM. Using a ready-to-use CT segmentation model, they delineated cardiac substructures from CT scans and calculated various quantification metrics, including the target-to-background ratio (TBR), cardiac pyrophosphate activity (CPA), and volume of involvement (VOI) from the registered SPECT images. They reported that all metrics achieved a diagnostic accuracy with an AUC of more than 0.98. Moreover, calculated parameters were significantly associated with cardiovascular death and heart failure hospitalization, demonstrating their potential in predicting clinical outcomes for ATTR-CM patients.

The current study inherently bears some limitations, including the scoring models, which are divided into 0 and 1 vs. 2 and 3 grades. This was due to the limited number of cases in different classes and a highly unbalanced training dataset for four-class classifications. The number of cases diagnosed as class #1 according to Perugini score was very limited in all the datasets included for training and testing our models. We attempted to generate simulated pseudo-planar images mimicking wide ranges of activity uptake in all four classes for training the segmentation model. However, the classification models with highly imbalanced data were less reliable. Due to the highly imbalanced nature of the four classes, we defined two classification tasks, including the detection task, where images in class #0 were separated from images in class #1, #2, or #3 (grade 0 vs. grades 1, 2, and 3), and the severity scoring task, to separate images in classes #0 and #1 from classes #2 and #3 (0 and 1 vs. grades 2 and 3). Human experts evaluation would have strengthened this work. However, we lack the resources to carry this evaluation owing to the high clinical workload. Although the same approach was taken in other studies, future research should focus on providing a four-grade scoring system. This might be addressed using synthetic data by generative models or simulating different classes in the training dataset. Moreover, we evaluated the model’s performance on one large dataset without ground truth for ATTR-CM, and only focused on positive cases. Future studies should evaluate the model’s performance prospectively and assess its impact on the diagnosis and prognosis of ATTR-CM patients. In our study, we did not evaluate the impact of the diagnostic model on patient outcomes. However, we have made the model publicly available. In future work, the provided models could be tested by other researchers or clinical centers on their own datasets to assess the relationship between model predictions and clinical outcomes, such as patient survival and heart failure hospitalization. The aim of this study was to develop an automated pipeline for detecting cardiac amyloidosis rather than focusing on comparisons with human experts. Future studies should compare the model’s performance with human observers to evaluate its added value in clinical practice. Finally, all ground truth for the ATTR-CM was defined visually by nuclear medicine physicians. This should be considered for cases where, even with negative scintigraphy, the presence of ATTR-CM is possible, and a biopsy is required. Our proposed framework offers several clinically valuable applications that enhance diagnostic support in various settings. It can be used as a screening tool to identify ATTR-CM in different scans, such as bone scintigraphy scans for benign or malignant bone diseases, that may otherwise be overlooked by clinicians not actively searching for ATTR-CM. The proposed algorithm also could help in reducing inter- and intra-observer variability, ensuring more consistent and reliable diagnoses. Furthermore, it has the potential to be applied in large-scale screening, both retrospectively and prospectively, enabling the detection of ATTR-CM from large datasets of bone scintigraphy images. All developed models and code for inference are available on our GitHub repository (https://github.com/YazdanSalimi/Cardicac-Amyloidosis).

## Conclusion

In the current study, we developed and evaluated a fully automated pipeline to detect and score ATTR-CM using multi-tracer, multi-scanner, and multi-center datasets, achieving high performance on TB images. Fully automatic detection and scoring of ATTR-CM could decrease inter- and intra-observer variability, and potentially detect more ATTR-CM cases. This fully automated pipeline could lead to timely and accurate diagnosis, ultimately improving patient outcomes.

## Electronic supplementary material

Below is the link to the electronic supplementary material.

Supplementary Material 1

## Data Availability

The data used in this work is not available.
