# Supplementary material for: Artificial intelligence-based cardiac transthyretin amyloidosis detection and scoring in scintigraphy imaging: multi-tracer, multi-scanner, and multi-center development and evaluation study
Source: Eur J Nucl Med Mol Imaging. 2025 Feb 5;52(7):2513–28. doi: 10.1007/s00259-025-07117-1 (PMC12119773; doi:10.1007/s00259-025-07117-1)
Supplement: Supplementary file 1 — Supplementary Material 1 [file 259_2025_7117_MOESM1_ESM.pdf]

Supplementary material

In the current study, we employed 6 different datasets (12 scanners) for various tasks and purposes including dataset #1, ^{99m}TC-MDP bone SPECT/CT images for training the 2D-planar segmentation and localization models; dataset #2, ^{99}TC-DPD bone scintigraphy images of patients suspected of ATTR for training the classification and grading models in a; dataset #3 (^{99m}TC-HDP), #4(^{99m}TC-PYP), and #5(^{99m}TC-DPD) patients suspected for cardiac amyloidosis as external evaluation from different centers. And finally, dataset #6, a large cohort of ^{99m}TC-MDP images without any label for ATTR-CM used for testing the capability of our model in a screening task. Two datasets, #1 and #2, were used to train our models, and four other datasets were used for externally validating our methodologies. The details of the included datasets are summarized below.

Training and internal validation data (#1 and #2)

This part included 233 total body planar (TB) images from 216 patients injected with TC-DPD and referred to roll-out cardiac involvement of ATTR at Geneva University Hospital (HUG). The delayed anterior/posterior total body planar images were acquired three hours after IV injection of ^{99m}TC-DPD. Data from three different scanners of Phillips BrightView, Siemens Symbia T6, Siemens Symbia T16 were included. Clinical reports, which were consensus between two physicians, were collected, and the information about Perugini's grade was introduced by Perugini et al. (Perugini, Guidalotti et al. 2005) from zero to three in four classes were recorded for each image. Grade #0 with no uptake in the myocardium, grade #1 for minimal uptake in myocardium less than bone tissues, grade #2 for uptake same as bone in the myocardium, and lastly, grade #3 for uptakes higher than bones in the myocardium.

From 233 images in dataset #1 the data distribution was as grade #0: 123, grade #1: 8, grade #2: 21, and grade #3: 81 images.

Dataset #2

This dataset, containing 93 multi-FOV SPECTs, was used to train the 2D-planar segmentation models acquired using a GE NM870 scanner at HUG. These patients were referred for evaluation of various bone pathologies, such as osteomyelitis and malignancies.

External Validation dataset (#3 to #6)

Dataset#3

The first external validation test set consisted of 39 TB planar images collected from a private imaging center inside Switzerland. These patients were injected with a ^{99}Tc-HDP tracer. As with Dataset #1, the physician reports were evaluated, and the Perugini grade was recorded. This dataset was acquired using a Phillips scanner. The data distribution was class #0: 19, class #1: 3, class #2: 3, and class #3: 14 cases for this dataset.

Dataset #4

A total number of 132 spot planar images acquired by three different scanners were collected. The Perugini score was read from the report written by a nuclear medicine physician, recorded, and used as the reference label. The important note about this dataset is the image acquisition mode, which was different from the training data. The training data was total body continuous bed motion acquisition, while this dataset was done using fixed bed position spot mage using different voxel spacing and acquisition modes. We included these images to test the robustness of our models against different acquisition modes. These patients were injected with a ^{99}Tc-PYP tracer. The data distribution was class #0: 29, class #1: 6, class #2: 14, and class #3: 4 cases for this dataset.

Dataset #5

This dataset contains 126 images collected from a Bern University Hospital injected with ^{99}Tc-DPD. The Perugini scores were collected in the same way as dataset #1, and our model tested against the physician-reported values as a reference. It should be mentioned that due to privacy issues, the trained models and inference code were shared with this center, and the inference happened offline in a real clinical scenario while the anonymized dataset was collected for other external centers. The data distribution was class #0: 51, class #1: 1, class #2: 19, and class #3: 54 cases for this dataset.

Supplementary figure 1 shows an example of images with incomplete segmentation but acceptable bounding box (left) and two rejected bounding boxes in our study (right).

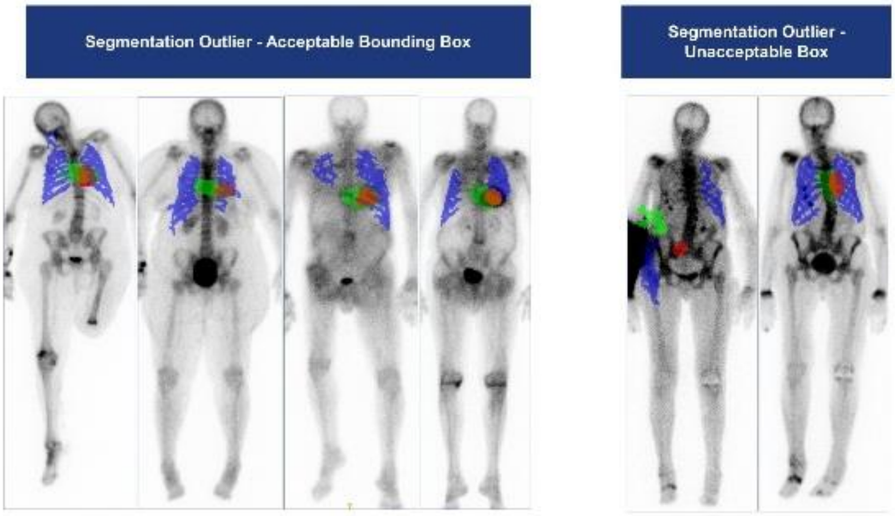

Supplementary figure 1. Segmentation outliers with acceptable and unacceptable bounding boxes were detected on the TB planar image.

Supplementary figure 2 shows the confusion matrixes for internal three-fold validation separated by crop strategies.

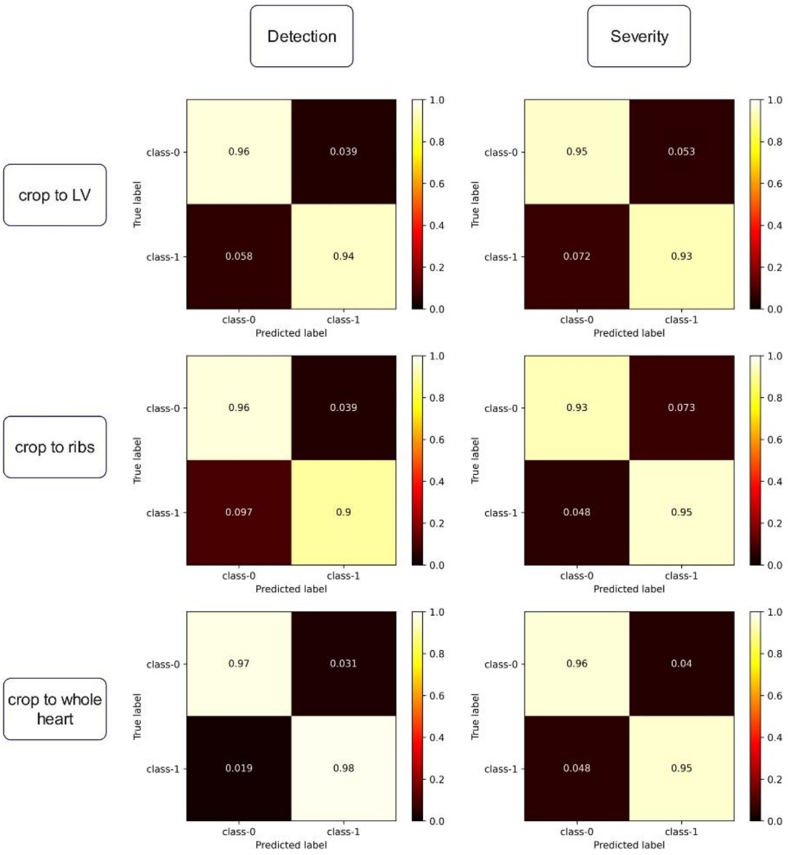

Supplementary figure 2. Confusion matrix images for internal three-fold cross validation.

Supplementary figure 3 shows the confusion matrixes for internal external validation test sets.

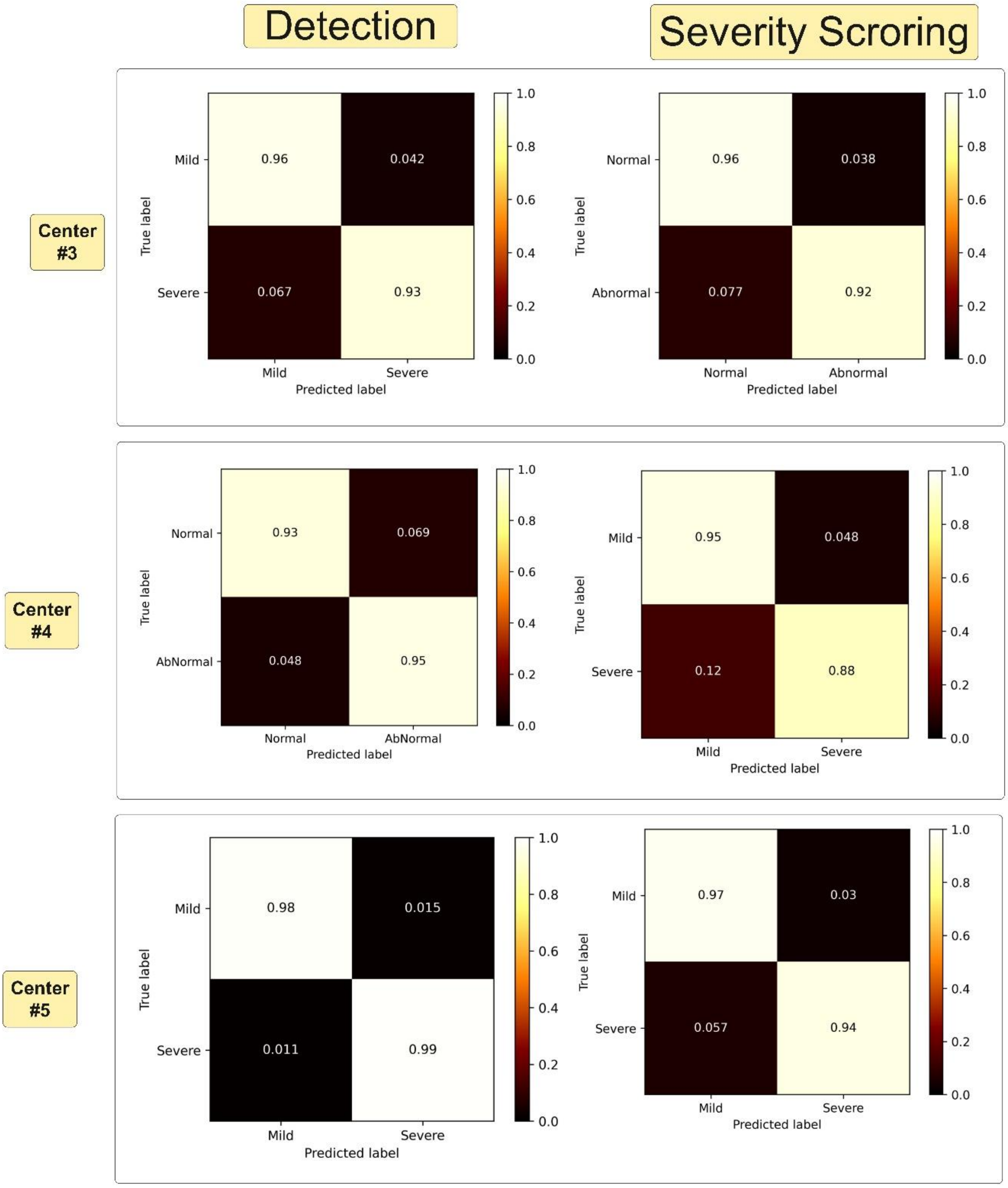

Supplementary figure 3. Confusion matrix images for external validation centers.

Supplementary figure 4 shows the images generated for explainable data augmentation to have a reliable segmentation model.

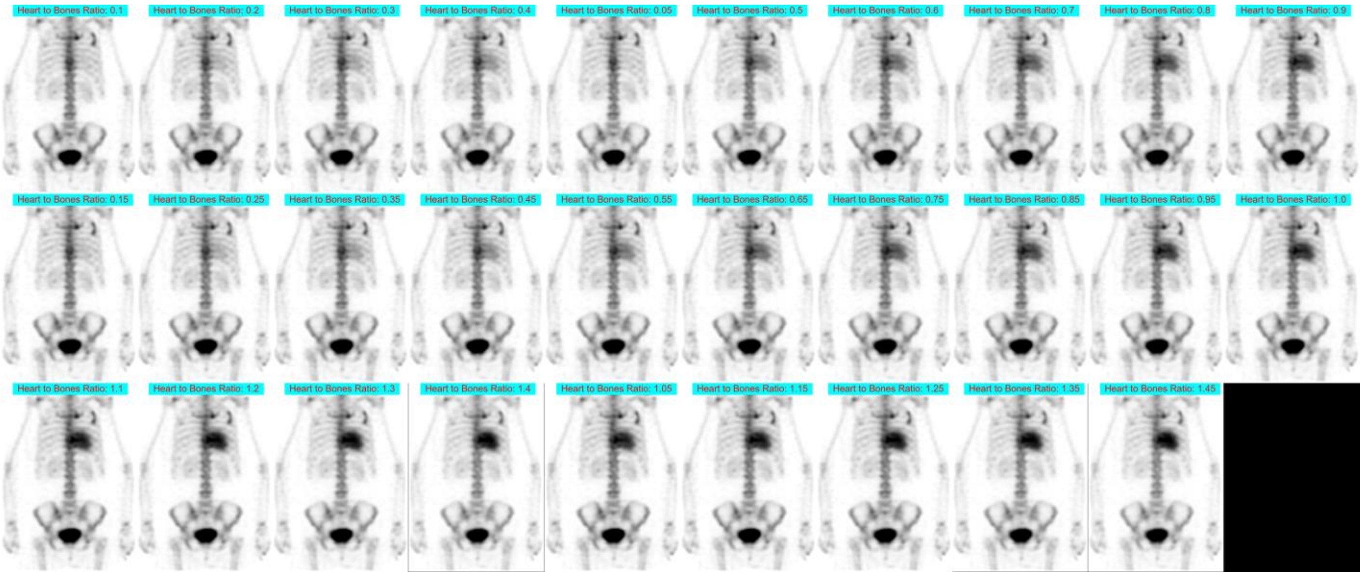

Supplementary Figure 4. The pseudo-planar images augmented through replacing the cardiac area with a random uptake of radiotracer to mimic different levels of ATTR pathologic planar images. The title of each image shows the ratio between the added uptake in the cardiac region versus the average uptake in the bones. i.e. heart to bone ratio of 1 shows an average uptake equal to bones in the cardiac area.

Supplementary figure 5 presents the total body and cropped images to ribs, LV, and whole heart visualized for the external dataset #3. The cropped areas were visualized and scored in a binary way to be accepted or rejected. As presented in the image with the red box, LV segmentation model failed on three of these and the ribs segmentation model failed in one case.

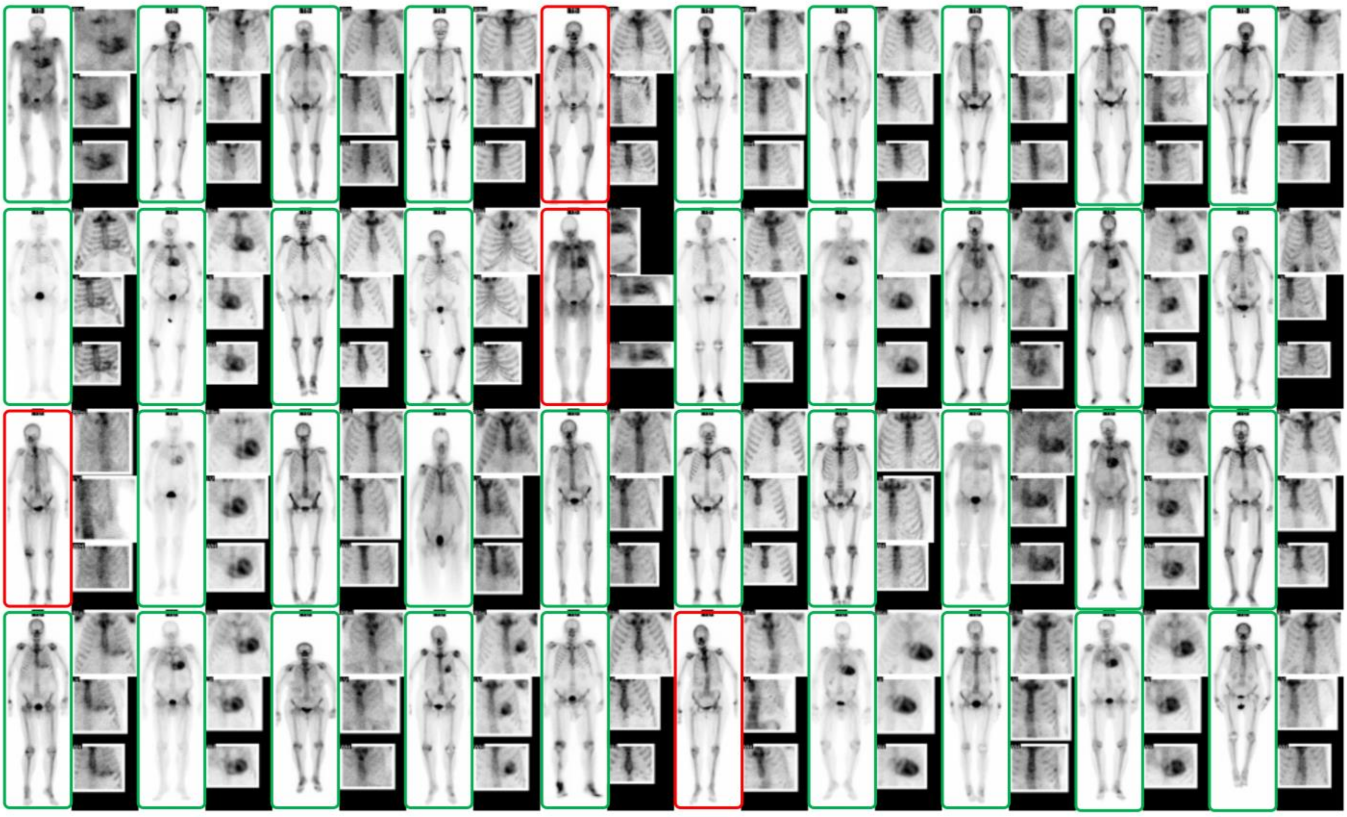

Supplementary figure 5. Total body image and the cropped region detected automatically using the segmentation model for ribs, LV, and whole heart. A green line around a case means accepted region detection whereas a red one reflects cases where at least one of the segmentation models failed.

References

Perugini E. et al. (2005). "Noninvasive etiologic diagnosis of cardiac amyloidosis using 99mTc-3,3-diphosphono-1,2-propanodicarboxylic acid scintigraphy." J Am Coll Cardiol **46**(6): 1076-1084.
